# Exercise to Counteract Alzheimer’s Disease: What Do Fluid Biomarkers Say?

**DOI:** 10.3390/ijms25136951

**Published:** 2024-06-25

**Authors:** Roberto Bonanni, Ida Cariati, Pierangelo Cifelli, Claudio Frank, Giuseppe Annino, Virginia Tancredi, Giovanna D’Arcangelo

**Affiliations:** 1 Department of Biomedicine and Prevention, “Tor Vergata” University of Rome, 00133 Rome, Italy; roberto.bonanni1288@gmail.com; 2 Department of Systems Medicine, “Tor Vergata” University of Rome, 00133 Rome, Italy; ida.cariati@uniroma2.it (I.C.); tancredi@uniroma2.it (V.T.); giovanna.darcangelo@uniroma2.it (G.D.); 3 Department of Applied Clinical and Biotechnological Sciences, University of L’Aquila, 67100 L’Aquila, Italy; pierangelo.cifelli@univaq.it; 4 UniCamillus-Saint Camillus International University of Health Sciences, 00131 Rome, Italy; claudio.frank@unicamillus.org; 5 Centre of Space Bio-Medicine, “Tor Vergata” University of Rome, 00133 Rome, Italy; 6 Sports Engineering Laboratory, Department of Industrial Engineering, “Tor Vergata” University of Rome, 00133 Rome, Italy

**Keywords:** neurodegeneration, Alzheimer’s disease, amyloid aggregates, fluid biomarkers, physiology, exercise, performance, cognitive function

## Abstract

Neurodegenerative diseases (NDs) represent an unsolved problem to date with an ever-increasing population incidence. Particularly, Alzheimer’s disease (AD) is the most widespread ND characterized by an accumulation of amyloid aggregates of beta-amyloid (Aβ) and Tau proteins that lead to neuronal death and subsequent cognitive decline. Although neuroimaging techniques are needed to diagnose AD, the investigation of biomarkers within body fluids could provide important information on neurodegeneration. Indeed, as there is no definitive solution for AD, the monitoring of these biomarkers is of strategic importance as they are useful for both diagnosing AD and assessing the progression of the neurodegenerative state. In this context, exercise is known to be an effective non-pharmacological management strategy for AD that can counteract cognitive decline and neurodegeneration. However, investigation of the concentration of fluid biomarkers in AD patients undergoing exercise protocols has led to unclear and often conflicting results, suggesting the need to clarify the role of exercise in modulating fluid biomarkers in AD. Therefore, this critical literature review aims to gather evidence on the main fluid biomarkers of AD and the modulatory effects of exercise to clarify the efficacy and usefulness of this non-pharmacological strategy in counteracting neurodegeneration in AD.

## 1. Introduction

Neurodegenerative diseases (NDs) represent a major cause of disability and mortality worldwide, with an economic and social impact, especially in the elderly population that is constantly growing [1]. These cognitive disorders are characterized by extremely complex pathological mechanisms that are not yet fully understood. Nevertheless, all NDs share some common events, including progressive neuronal death and altered synaptic transmission and plasticity, with severe repercussions on higher cognitive functions, such as memory and learning, and an individual’s motor skills [2,3,4,5].

NDs are caused by a group of unrelated proteins with some common characteristics, such as the tendency to form insoluble aggregates of different sizes, and neurotoxicity [6]. Between them, Alzheimer’s disease (AD) is undoubtedly the best-known and most widespread ND disease, in which amyloid aggregates formed by the beta-amyloid (Aβ) protein and the hyperphosphorylated Tau protein result in an initial short-term memory loss that then progresses into typical dementia that characterizes this disease [7]. In a recent report published in the Lancet, Scheltens et al. reported that, by 2050, the prevalence of AD-related dementia will double in Europe and triple worldwide, highlighting the need to develop strategies to counter its progression [8].

Interestingly, numerous synaptic and neuronal integrity proteins can be detected in the body fluids of AD and other ND patients, highlighting their potential role as biomarkers of neurodegeneration [9,10,11]. Early detection of such biomarkers in cerebrospinal fluid (CSF) and plasma could facilitate both the early diagnosis of neurodegenerative disorders and the initiation of pharmacological treatment, which will be more effective if undertaken in the early stages of the diseases [12]. Furthermore, such fluid biomarkers in patients’ CSF could provide valuable information on disease progression and actual treatment efficacy, thus acquiring diagnostic and prognostic value [13]. However, despite the enormous efforts of research in this field, to date, there is still no solution capable of definitively defeating neurodegenerative disorders, highlighting the need to adopt strategies to slow down the ND’s progression, attenuating both cognitive and motor symptoms [14].

In this context, exercise is an excellent tool for preventing the onset of NDs and counteracting its progression [15]. Indeed, numerous studies have demonstrated the effectiveness of exercise in limiting cognitive decline in AD patients [16]. However, investigations on fluid biomarkers of neurodegeneration often report conflicting results, suggesting the need for further knowledge on the ability of exercise to counteract neurodegeneration in AD. Therefore, this literature review aims to i) examine the role of biomarkers of synaptic and neuronal integrity as potential diagnostic factors of AD and ii) collect evidence to evaluate the efficacy of exercise as a valid tool to counteract neurodegeneration in AD by modulating the concentrations of these biomarkers in body fluids.

## 2. AD: A Brief Overview of Pathogenesis

Some biochemical and biophysical events are underlying the pathogenesis of several NDs [17]. Particularly, the formation of pores in neuronal membranes leading to an imbalance in calcium homeostasis has been suggested as a common mechanism in many amyloid storage neurodegenerative disorders. The result is vesicular depletion with neurotransmitter reduction, impaired synaptic transmission, and neuronal death [18]. Although this sequence of events is common to several diseases, the molecular mechanisms and genetic determinants leading to the onset of AD require special discussion.

### AD

Most cases of AD are sporadic and late-onset, making it extremely difficult to identify the underlying causes of the genesis of AD-related dementia [19]. In fact, more than 20 genetic risk factors have been identified as being responsible for the onset of AD [20]. Among these, the *APOE* gene represents the largest single risk factor, as demonstrated by the increased likelihood of developing AD in carriers of the ε4 allele, particularly homozygotes [21]. On the other hand, mutations in the genes encoding for amyloid precursor protein (APP), presenilin 1 (PSEN1), and presenilin 2 (PSEN2) have been associated with a rare familial form and early onset of AD [22]. Specifically, under non-amylogenic conditions, APP, a transmembrane protein with extracellular domains, undergoes cleavage by the enzyme α-secretase, producing soluble, non-pathogenic peptide fragments that undergo further cleavage by the enzyme γ-secretase. In AD, the so-called amyloidogenic pathway is active, in which APP is first cleaved by the β-secretase enzyme (BACE) and then by γ-secretase, with the formation of Aβ peptides that aggregate, leading to the formation of neurotoxic prefibrillar oligomers (PFOs) [23]. It is noteworthy that the neurotoxicity of Aβ peptides varies considerably depending on the fragment formed by the action of γ-secretase, as the Aβ_1–40_ peptide is characterized by significantly lower toxicity than the Aβ_1–42_ fragment, which is associated with early-onset familial AD [24].

Importantly, AD is characterized by the deregulation of several kinases and phosphatases that act on the Tau protein, resulting in its hyperphosphorylation [25]. The Tau protein has a microtubule-binding domain that allows it to co-assemble with tubulin to form mature, stable microtubules [26]. In AD, the altered action of both proline-directed kinases, such as glycogen synthase kinase-3 (GSK3) and cyclin-dependent protein kinase-5 (CDK5), and mitogen-activated kinases (MAPKs), results in hyperphosphorylation of Tau, which dissociates from the microtubule and tends to aggregate to form characteristic neurofibrillary tangles (NFTs) [27]. As a result, microtubules become unstable and disassemble, leading to cytoskeletal degeneration with a deficit in vesicular transport [28]. The impairment of this process, which is fundamental for the proper functioning of neurons, leads to the formation of Aβ accumulations in the axon terminal, causing the failure of synaptic transmission [23]. Furthermore, in axon terminals, calcium deregulation induced by the amyloid pores formed by Aβ oligomers promotes constitutive neurotransmitter release in the inter-synaptic cleft and inevitable vesicular depletion. All these events, combined with the alteration of the intracellular redox state due to mitochondrial dysfunction by Aβ, result in apoptotic neuronal death [29].

It is noteworthy that autophagy and mitophagy, evolutionarily conserved cellular events in eukaryotes, are found to be defective in AD and promote the accumulation of Aβ and Tau aggregates, with dramatic consequences on neuronal health [30,31]. Indeed, these processes represent the main pathway by which cells degrade dysfunctional organelles and protein aggregates and play an essential role in neuronal homeostasis, being terminally differentiated and non-substitutable cells [32]. In this context, Reddy and colleagues reported that the accumulation of mutated Aβ and APP in the hippocampal cell line HT22 drastically impairs mitochondrial function and biogenesis, reducing the expression of the dendritic proteins, including microtubule-associated protein 2 (MAP2), and synaptic proteins, including synaptophysin and postsynaptic density protein 95 (PSD-95), as well as altering autophagy and mitophagy processes, resulting in neuronal dysfunction and impaired cell viability [33]. Thus, the accumulation of amyloid deposits in AD is responsible for cognitive impairment by altering several fundamental cellular processes.

The presence of cognitive and behavioral symptoms is the basis for the diagnosis of AD, as it has been reported that among individuals aged 70 years or older, only 20–40% have biomarkers typical of AD, highlighting the inadequacy of autopsy findings for its diagnosis [34]. However, detecting neuronal and synaptic integrity proteins in body fluids could facilitate an accurate and timely diagnosis, which is crucial for the optimal management of AD patients. Furthermore, monitoring the concentrations of such biomarkers in body fluids could provide valuable information regarding the staging of AD and the efficacy of therapeutic interventions. Finally, although the extraordinary therapeutic power of exercise in AD patients is widely known and well documented, the analysis of biomarkers detected in the fluids of AD patients undergoing exercise programs needs further investigation to verify both the efficacy of specific exercise protocols and the diagnostic and/or prognostic power of individual biomarkers.

## 3. Fluid Biomarkers as Diagnostic and Prognostic Factors of AD

Generally, by the time neurodegenerative diseases are diagnosed, the damage caused by neurodegeneration is already severe [34]. To date, the most widely used approach for diagnosing AD involves the use of neuroimaging techniques such as magnetic resonance imaging (MRI), positron emission tomography (PET), and single-photon emission computed tomography (SPECT). These techniques can provide valuable information on structural and functional changes in affected brain areas, helping to diagnose neurodegenerative disorders in the prodromal stages [35]. However, although neuroimaging techniques represent the gold standard for diagnosing AD, a good deal of biological research in this field is focused on identifying biomarkers, detectable in body fluids, as potential diagnostic and prognostic factors of AD.

**Figure 1:**
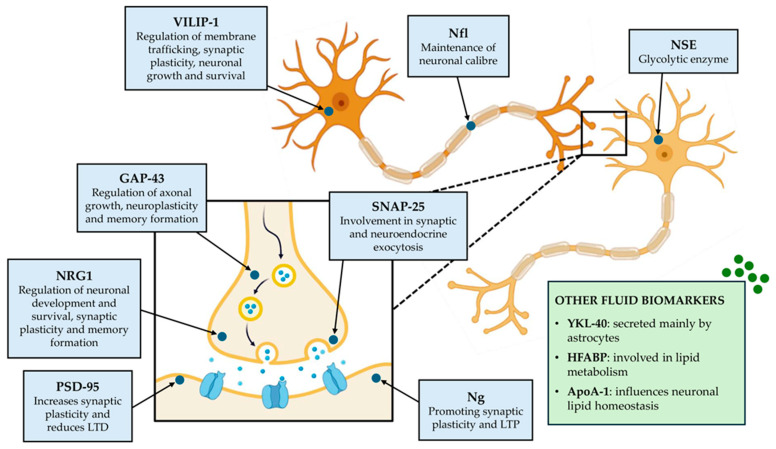
A schematic representation of the main fluid biomarkers involved in Alzheimer’s disease (AD). In the cytoplasm of neurons, neuron-specific enolase (NSE) with glycolytic enzymatic action and visinin-like protein 1 (VILIP-1), which regulates membrane transport, synaptic plasticity, as well as neuronal growth and survival, are localized. Neurofilament light chain (Nfl) is an intermediate filament protein localized in the axon, which controls the maintenance of the neuronal caliber. Other proteins are in the cytoplasm of the presynaptic neuron: growth-associated protein 43 (GAP-43), involved in axonal growth, neuroplasticity, and memory formation; neuregulin 1 (NRG1) regulates neuronal development and survival, synaptic plasticity, and memory modulation; synaptosomal-associated protein 25 (SNAP-25) is responsible for synaptic and neuroendocrine exocytosis. At the level of the postsynaptic membrane, postsynaptic density protein 95 (PSD-95), which increases synaptic plasticity and reduces long-term depression (LTD), and neurogranin (Ng), which promotes synaptic plasticity and long-term potentiation (LTP), are localized. Other fluid biomarkers associated with the pathogenesis of AD, but not localized in neurons, include chitinase 3-like protein 1 or human cartilage glycoprotein 39 (YKL-40) secreted mainly by astrocytes, heart fatty acid binding protein (HFABP) with a role in lipid metabolism, and apolipoprotein A1 (ApoA-1) that influences neuronal lipid homeostasis.

Figure 1 summarizes the main fluid biomarkers discussed below, focusing on location and function.

### 3.1. Biomarkers of Neurodegeneration in the CSF of AD Patients

Although numerous molecular actors are involved in AD pathogenesis that could provide valuable information on the diagnosis or progression of the disease, some of these indicate a condition of neuronal and/or synaptic damage, thus acting as indicators of neurodegeneration [36].

The finding of Aβ in CSF dates to 1992, when Seubert and colleagues demonstrated that the peptide responsible for AD was produced and released both in vivo and in vitro, laying the foundation for the development of diagnostic tests based on the presence of amyloid in CSF [37]. Following the identification of the peptide Aβ_42_ as the main peptide responsible for neurodegeneration in AD, numerous scientists attempted to positively correlate the amount of this peptide in CSF with cognitive impairment, but with poor results. Indeed, numerous reports have shown that the CSF of AD patients is characterized by low levels of Aβ_42_ [38,39]. This reduction could be explained by the aggregative behavior of this peptide, as Fagan et al. demonstrated the existence of an inverse relationship between low Aβ_42_ levels and the presence of amyloid plaques in AD patients [40]. This association has been confirmed by several studies, with a concordance value of 90%, emerging as a potential preclinical biomarker of AD [41]. Other Aβ species have also been found in the CSF of AD patients, such as the peptide Aβ_40_. Particularly, the Aβ_42_/Aβ_40_ ratio, besides offering higher performance in the identification of AD, shows better concordance with PET positivity for amyloid [42].

The presence of the Tau protein in the CSF is also considered a biomarker of AD. Particularly, the total Tau (T-Tau) protein has been proposed as a marker of the severity of neurodegeneration, while the phosphorylated Tau (P-Tau) protein in residues 231, 181, or 199 can discriminate AD from other NDs [43]. Furthermore, P-Tau217 has been proposed as a potential diagnostic and prognostic biomarker of AD, with a higher sensitivity than P-Tau181, as its levels are significantly increased in PET-positive Aβ mild cognitive impairment (MCI) patients [44]. In general, in the context of AD, the presence of T-Tau and P-Tau in the CSF can predict faster disease progression, highlighting their role as biomarkers of AD [45,46,47].

Neurogranin (Ng) is a postsynaptic protein involved in synaptic plasticity and long-term potentiation (LTP), processes underlying memory formation, whose increases in the CSF could reflect marked synaptic loss and profound structural alterations [48]. In this regard, Mavroudis et al. conducted a systematic literature review with a meta-analysis comparing the results of studies that analyzed the presence of Ng in the CSF in different NDs. Significantly higher levels of Ng were found in AD patients compared to patients with MCI, frontotemporal dementia (FTD), and other NDs, suggesting its role as a reliable diagnostic biomarker for the diagnosis of AD as well as for discrimination against other disorders [49]. On the other hand, Willemse et al. evaluated Ng levels in the CSF of a dementia cohort consisting of AD patients, AD patients with high T-Tau, Creutzfeldt–Jakob disease (CJD) patients, and non-AD subjects and controls, concluding that Ng in the CSF represents a biomarker of synaptic degeneration, closely related to Tau but not specific to AD [50].

Neuron-specific enolase (NSE) is a neuronal glycolytic enzyme that indicates the presence of acute or prolonged neuronal damage [51]. Its role as a biomarker of AD dates back to 1995 when Parnetti and colleagues found a correlation between NSE levels in the CSF and the severity of cognitive deficits [52]. Subsequently, Palumbo et al. conducted a comparative study of AD patients and healthy controls to measure concentrations of NSE, Aβ_42,_ and T-Tau in CSF, finding a significant increase in NSE and T-Tau and a significant decrease in Aβ_42_. Interestingly, a direct correlation of NSE with T-Tau and an inverse correlation with Aβ_42_ was found, suggesting NSE as a specific marker of AD being correlated with major biomarkers [53]. In agreement, Schmidt and colleagues found significantly elevated levels of NSE in the CSF of AD patients compared to the control group, confirming its role as a biomarker of cognitive impairment and its direct correlation with T-Tau and P-Tau [51]. Finally, Katayama et al. conducted a systematic review with a meta-analysis to investigate the utility of NSE levels in CSF as a biomarker of some NDs. Significantly elevated levels of NSE were observed in the CSF of AD patients, although this biomarker can also be found in the CSF of patients with Parkinson’s disease (PD), concluding that NSE may be a useful indicator of neurodegeneration in these disorders [54].

The neurofilament light chain (Nfl) is a neuronal cytoplasmic protein that is highly expressed in myelinated large-caliber axons, the levels of which increase in the CSF proportionally to the degree of axonal damage. Therefore, this protein could reliably play the role of a biomarker of neurodegeneration in a wide variety of neurological disorders, including inflammatory, neurodegenerative, traumatic, and cerebrovascular diseases [55]. In this regard, in 2019, Bridel and colleagues published in JAMA the results of a systematic literature review with a meta-analysis on the diagnostic value of Nfl in certain neurological disorders. The authors found that Nfl levels in the CSF were significantly higher in almost all neurodegenerative disorders studied, indicating its potential role as a marker in neuroaxonal degeneration [56]. More recently, Leckey and colleagues found no significant changes in Nfl levels in the CSF of AD patients, behavioral variant of FTD (bvFTD) patients, corticobasal syndrome (CBS) patients, dementia with Lewy Bodies (DLB) patients, Huntington’s disease (HD) patients, multiple sclerosis patients, and patients with semantic dementia, confirming its non-specificity for neurodegeneration in AD [57].

Visinin-like protein 1 (VILIP-1), which belongs to the group of neuronal calcium sensor proteins (NCS), performs several crucial functions in the central nervous system (CNS), regulating ion channels, membrane trafficking, synaptic plasticity, neuronal growth, and survival [58]. This protein is considered an emerging biomarker that can aid in the early diagnosis of AD, as deregulation of calcium homeostasis results in axonal degeneration and release of VILIP-1 into the CSF [59]. Indeed, a comparison of VILIP-1 levels in the CFS of AD patients with those of healthy subjects and MCI patients showed that this protein was significantly more represented in AD patients. In addition, VILIP-1 levels correlated with elevated T-Tau levels and reduced Aβ_42_ levels, confirming its role as an effective diagnostic biomarker of AD [59,60].

Interestingly, altered lipid metabolism is an event that characterizes AD and leads to changes in membrane composition and fluidity, contributing to neuronal dysfunction [61]. Therefore, lipid-binding proteins could play an important role in the pathogenesis of AD as predictors of neuronal plasma membrane modifications leading to neuronal deterioration [62,63]. In this context, heart fatty acid binding protein (HFABP) has been suggested as a diagnostic and prognostic biomarker in the early stages of AD [64]. Indeed, in 2013, Desikan et al. demonstrated that high levels of HFABP in CSF, concomitantly with low levels of Aβ_42_, were associated with brain atrophy of selectively affected areas in the early stages of AD. Importantly, the authors reported that HFABP is not simply a generalized marker of neuronal damage, but high levels of HFABP in CSF may reflect the deregulation of lipid homeostasis in the CNS [65]. This observation suggests a crucial role of CNS lipids in AD pathogenesis that could reflect the involvement of proteins responsible for lipid metabolism. Indeed, reduced levels of apolipoprotein A1 (ApoA-1) have been observed in the CSF of AD patients compared to MCI patients and healthy controls, suggesting its potential role as a fluid biomarker for AD diagnosis [66]. Moreover, this protein does not contribute to neuronal integrity as it is synthesized in the liver and intestine and is responsible for transporting excess cholesterol from peripheral tissues to the liver. However, ApoA-1 has been suggested to enter the brain and influence neuronal lipid homeostasis [67]. Particularly, Slot et al. measured ApoA-1 levels in the CSF of elderly people with cognitive decline (SCD) and MCI, detecting increased levels of the protein in *APOE* ε4 carriers with cognitive decline and confirming its role as a biomarker in the early stages of AD [68]. Other evidence has shown that reduced levels of ApoA-1 in the CSF are associated with AD, although it is not entirely clear whether this biomarker can be considered specific for AD or whether it signals the presence of neuronal damage [67].

Growth-associated protein 43 (GAP-43) is a protein found on the cytoplasmic side of the presynaptic membrane [65] and is involved in axonal growth, neuroplasticity, and memory formation [69]. This protein is abundantly expressed in the cerebellum, neocortex, entorhinal cortex, hippocampus, olfactory bulb, and retinal cells and has been suggested as a biomarker of synaptic dysfunction, being abundantly present in the CSF of AD patients [70,71,72,73]. Specifically, Franzmeier and colleagues observed that GAP-43 levels in the CSF of AD patients were associated with a more rapid accumulation of Aβ-related Tau. In other words, the effect of Aβ on Tau deposition was greater in the presence of high levels of GAP-43 in the CSF, highlighting the role of this presynaptic protein as a biomarker of synaptic dysfunction in AD [74].

Chitinase 3-like protein 1 or human cartilage glycoprotein 39 (YKL-40) is a chitin-binding lectin and belongs to the glycosyl hydrolase 18 family [75]. It has been indicated as a marker of neuroinflammation that can facilitate the diagnosis of AD, as demonstrated by increased levels of this protein in the CSF of AD patients compared to healthy controls [76,77]. Interestingly, YKL-40 could represent a valid tool for predicting the conversion of MCI to AD, as differences were found in CSF between the two patient cohorts [78].

Another protein that could play the role of a biomarker of neurodegeneration is PSD-95, which is known to bind to the C-terminal domain of glutamate N-Methyl-D-Aspartate Receptors (NMDARs), affecting synaptic transmission and plasticity. Indeed, up-regulation of PSD-95 has been reported to enhance synaptic transmission and inhibit long-term depression (LTD) [79]. Furthermore, the brain tissue of AD patients is known to be characterized by reduced expression of PSD-95, suggesting its potential to signal neural damage in AD pathogenesis [80]. In this context, Kivisäkk and colleagues investigated the role of PSD-95 as a potential fluid biomarker of AD by comparing protein levels in the CSF of AD patients with those of other patients with different neurological conditions. The authors found high levels of PSD-95 in all types of patients, suggesting that this protein may be a valid marker of non-highly specific synaptic damage in AD [81].

Synaptosomal-associated protein 25 (SNAP-25) is widely distributed in the brain, performing crucial functions such as synaptic and neuroendocrine exocytosis [82]. Several authors have investigated the role of SNAP-25 as a fluid biomarker, finding the existence of a positive relationship with Aβ pathology [83,84]. Notably, in 2018, Wang and colleagues published results of a comparison of SNAP-25 levels in the CSF of patients with MCI, dementia, mild AD, and normal cognition, in carriers and non-carriers of *APOE* ε4. The authors showed that SNAP-25 was more abundant in the CSF of AD and MCI patients and that, among MCI patients, SNAP-25 levels were higher in *APOE* ε4 carriers than non-carriers, suggesting the ability of this protein to indicate presynaptic degeneration preceding AD [85]. It is noteworthy that SNAP-25 levels were also increased in the CSF of cognitively normal elderly patients who were *APOE* ε4 carriers, indicating the existence of selective synaptic damage in these subjects compared to their non-carriers [86]. Finally, in 2022, Kivisäkk and colleagues detected significantly increased levels of SNAP-25 in AD patients compared to other NDs, suggesting its role as a potential AD-specific biomarker [81].

Neuregulin 1 (NRG1) is a neurotrophic factor that stimulates the release of gamma-aminobutyric acid (GABA) [87]. This pre-synaptic protein is cleaved by the enzyme BACE-1 and can activate the postsynaptic receptor tyrosine-protein kinase erbB4 (ErbB4), regulating neuronal processes such as development, synaptic plasticity, neuronal survival, and modulation of memory [88]. In the retrospective study by Mouton-Liger et al., which included a total of 162 subjects, NRG1 levels in the CSF of AD patients were significantly increased compared to controls and subjects with other neurological disorders, underlining the specificity of NRG1 to signal synaptic impairment typical of AD [88].

Overall, changes in the levels of these biomarkers in the CSF of AD patients could provide valuable support for early diagnosis, facilitating the identification of AD patients in the prodromal phase and the timely initiation of the treatment pathway.

Table 1 summarizes the main scientific evidence on the levels of biomarkers discussed in the text in the CSF of AD patients or patients with other NDs.

### 3.2. Plasma Biomarkers in AD

Although the diagnostic relevance of Aβ_40_ and Aβ_42_ in the CSF of AD patients has been documented, their presence at the plasma level would not appear as useful for diagnosing AD [89]. However, Nakamura et al. reported in Nature that APP/Aβ_42_ and Aβ_40_/Aβ_42_ ratios, detected in plasma through immunoprecipitation coupled to mass spectrometry, can predict amyloid accumulation in the brain, suggesting the diagnostic importance of these biomarkers in the AD pathogenesis [90]. In agreement, Cai and colleagues monitored the plasma concentration of Aβ_42_ in control subjects and in patients with preclinical AD, finding a slight reduction between the two cohorts. Importantly, plasma levels of the protein were significantly reduced in AD patients at follow-up, confirming Aβ_42_’s ability to predict the AD development 8 to 10 years before disease onset [91].

Regarding the role of tau in blood, Moscoso and colleagues reported that the presence of P-Tau in the plasma of AD patients is closely associated with Aβ deposition, neurodegeneration, cognitive decline, and disease progression [92]. Interestingly, plasma brain-derived tau (BD-Tau) level, rather than T-Tau, has been suggested to represent an AD-specific neurodegenerative biomarker associated with clinical disease severity, as demonstrated by the strong association between BD-Tau concentrations in plasma and CSF [93]. The high sensitivity of P-Tau, and particularly the phosphorylated form at residue 181, has also been documented by other authors, to the point of being considered an easy biomarker capable of predicting pathologies characterized by tau and Aβ accumulation, discriminating between AD and other NDs, as well as identifying AD in the clinical continuum [94,95]. Particularly, Janelizde and colleagues investigated the role of P-Tau181 in the plasma of AD patients, MCI patients, non-AD patients, and cognitively normal subjects, concluding that high plasma P-Tau181 can discriminate AD from other NDs. Therefore, the authors suggested a role for P-Tau181 as a non-invasive diagnostic and prognostic biomarker of AD [96]. In addition, Milà-Alomà et al. highlighted the prognostic importance of P-Tau231 and P-Tau217 being able to detect early brain changes associated with the presence of Aβ before the appearance of clinical signs of AD [97].

To test the utility of plasma Ng, De Vos and colleagues measured, by enzyme-linked immunosorbent assay (ELISA), the C-terminal portion of Ng in paired CSF and plasma samples of 29 controls compared to 29 MCI patients or those with dementia due to AD. Although the presence of Ng in the CSF confirmed its diagnostic power, as the increased concentration correlated positively with Tau levels, no differences were found at the plasma level between controls and AD patients, suggesting the unreliability of plasmatic Ng for diagnosing AD [98,99].

In 2023, Chatterjee et al. evaluated the presence of plasma AD biomarkers in correlation to PET positivity for Aβ, investigating, in a transversal manner, the variations in Aβ_1–42_/Aβ_1–40_, P-Tau181, glial fibrillary acidic protein (GFAP), and Nfl along the continuum of AD [100]. The authors found that Aβ-PET-positive patients with cognitive decline were characterized, compared to Aβ-PET-negative patients, by a lower Aβ_42_/Aβ_40_ ratio, an elevated P-Tau181 concentration, as well as increased Nfl levels. It is noteworthy that plasma Nfl levels were elevated in AD patients and patients with prodromal AD but not in those with preclinical AD, highlighting the prognostic potential of Nfl to predict AD progression [100]. This evidence agrees with what was previously shown by Baiardi et al., who analyzed the presence of biomarkers in plasma and CSF samples from patients with AD and other NDs. In addition to confirming the high diagnostic value of P-Tau181, the authors found that Nfl was more highly represented in the body fluids of ND patients compared to healthy controls, suggesting the ability of this biomarker to signal neuronal damage not specific to AD [101]. Importantly, although Cai and colleagues reported that increased plasma Nfl predicts the development of AD 8 to 10 years before the disease, it is unclear whether or not this biomarker is specific for AD [91].

A detailed investigation into the emerging AD marker role of serum VILIP-1 was conducted by Halbgebauer et al., who analyzed paired CSF and serum samples from patients with AD or other NDs. The concentration of VILIP-1 in CSF and serum was higher in AD patients than in controls, although higher concentrations were found in the fluids of CJD patients, suggesting a useful role of VILIP-1 in the differential diagnosis of AD [59].

Similar results were obtained by Steinacker and colleagues studying the potential of HFABP in the differential diagnosis of NDs [102]. The authors measured the concentration of this biomarker in the CSF and plasma of AD patients, CJD patients, DLB patients, and controls, finding increased levels in all groups with NDs compared to healthy controls. Interestingly, HFABP was more represented in the CSF of CJD patients and in the serum of DLB patients, suggesting the usefulness of this biomarker in the differential diagnosis of neurodegenerative disorders [102].

In agreement with observations conducted by analyzing CSF, reduced levels of ApoA-1 were also found in the plasma of AD patients compared to controls. It is noteworthy that such reduction appears to be associated with a greater risk of clinical progression to MCI and AD, probably because ApoA-1 appears to play a neuroprotective role on neurons by counteracting Aβ-induced neurodegeneration [68,103,104]. However, Slot et al. observed that the risk of clinical progression in subjects carrying *APOE* ε4 is associated with elevated levels of ApoA-1 in CSF but reduced levels in plasma, suggesting the need to clarify the role of ApoA-1 in the development of AD [68].

An important result was provided by Jia and colleagues, who investigated the presence of synaptic proteins in the CSF and in neuronal-derived exosomes isolated in the blood of AD patients, MCI patients, and healthy subjects. Interestingly, GAP-43, Ng, and SNAP-25 were increased in CSF and decreased in exosomes isolated from the blood of AD and MCI patients, suggesting a role for such exosomal biomarkers in distinguishing AD from MCI patients and in predicting AD 5 to 7 years before cognitive deterioration [99].

Choi et al. reported the importance of YKL-40 in plasma as a biomarker of AD and analyzed its levels in samples taken from AD patients, MCI patients, and control subjects [105]. A significant increase in the plasma concentration of YKL-40 was observed in patients with early AD, compared to the other experimental groups, suggesting the ability of this marker to highlight the severity of AD. Interestingly, plasma YKL-40 levels in patients with mild AD, but not in those with moderate or severe AD, correlated positively with cognitive assessment test results, highlighting its potential to signal the onset of cognitive symptoms of AD [105].

Regarding the role of NRG1 as a plasma biomarker of AD, Chang et al. found a higher concentration in samples taken from AD patients than in healthy individuals. It is noteworthy that AD patients were stratified into mild and moderate AD groups based on mini-mental status exam (MMSE) scores. A significant relationship was found between NRG1 levels and disease severity, as plasma concentrations of this biomarker were higher in the group with lower MMSE scores [106]. In agreement, Vrillon and colleagues reported the existence of a close association between increased plasma levels of NRG1, cognitive decline, and synaptic dysfunction, suggesting its role as a potential non-invasive biomarker for monitoring neuronal damage in AD [107].

Overall, this evidence suggests a salient role of plasma biomarkers in signaling neuronal damage associated with cognitive decline. Furthermore, investigating AD characteristics, in terms of disease severity and evolution, by means of a blood sample offers the possibility of acquiring relevant information on AD patients in a rapid and non-invasive manner. Importantly, plasma-level concentrations of fluid biomarkers could reflect the effect of a management course, highlighting the usefulness or ineffectiveness of a particular treatment.

Table 2 summarizes the main scientific evidence on the levels of biomarkers dis-cussed in the text in the plasma of AD patients or patients with other NDs.

## 4. Exercise in AD to Counteract Neurodegeneration

Exercise is a recommended non-pharmacological strategy for AD patients, but the effects of such an intervention on fluid biomarker concentrations are poorly supported. For this reason, we report below the main evidence in which the role of exercise in modulating AD fluid biomarker concentrations has been documented.

Several pieces of evidence have shown the benefits of exercise in AD patients, particularly on cognitive function and physical performance, facilitating the performance of activities of daily living [108,109,110]. From a molecular perspective, exercise can counteract AD progression by regulating processes such as neuronal apoptosis, intercellular communication, oxidative stress, mitochondrial autophagy, synaptic plasticity, and neurotoxicity [111]. The multiple benefits of exercise on the CNS make it an ideal strategy to prevent and/or counteract the cognitive decline and neurodegeneration that characterize NDs. However, current evidence points to the need for further studies both to determine the efficacy of exercise in modulating the expression of neurodegeneration biomarkers and to establish which type and exercise programs are most effective for the well-being of AD patients. Indeed, in 2017, Jensen and colleagues published the results of a randomized controlled trial (RCT) aimed at assessing the effects of exercise on biomarkers of neuronal and synaptic integrity [112]. In this trial, 51 AD patients were randomized into two groups, one undergoing 16 weeks of moderate-to-high aerobic exercise and one undergoing usual care as a control group. Before and after the intervention, CSF was taken to analyze the levels of Nfl, Ng, VILIP-1, and YKL-40 to compare the mean change from baseline between the exercise and control groups. The authors found no significant differences in the concentrations of the investigated biomarkers, concluding that moderate or high-intensity exercise does not modulate the concentration of neuronal integrity biomarkers in the CSF of AD patients [112]. However, in 2018, Law et al. examined the relationship between physical activity levels and the concentration of Aβ_42_ and tau in CSF in asymptomatic middle-aged adults at risk for AD [113]. In this study, 85 cognitively healthy middle-aged adults wore an accelerometer for one week to measure daily physical activity level and underwent lumbar puncture for CSF sampling. Neither light nor vigorous physical activity produced relevant changes in the concentration of Aβ_42_ and Tau. However, moderate-intensity physical activity promoted marked changes in the investigated biomarkers, as it was associated with increased levels of Aβ_42_ and a reduced ratio of both T-Tau/Aβ_42_ and P-Tau/Aβ_42_, indicating a favorable AD biomarker profile [113]. On the other hand, Sewell et al. studied the effects of 6 months of moderate- or high-intensity exercise in 99 cognitively normal older adults to assess possible changes in plasma levels of potential AD biomarkers, including Aβ_42_, P-Tau181, and Nfl. The authors observed no significant changes in the plasma levels of the biomarkers analyzed, suggesting the need for studies with longer follow-up periods to highlight any exercise-induced effects [114]. In contrast, Hou et al. evaluated the self-reported lifestyle of 1108 cognitively normal adults, of whom 161 were *APOE* ε4 carriers, and found an association between the daily practice of moderate-intensity physical activity and a significant reduction in P-Tau181 in the CSF [115]. In agreement, Yu and colleagues conducted a randomized trial of 26 older adults who were *APOE* ε4 carriers with mild-to-moderate AD dementia. Of these, 18 performed cycling exercises on a recumbent stationary cycle at moderate intensity 3 days/week for 6 months, while the other 8 older adults performed low-intensity stretching exercises for the same period. The authors observed a reduction in plasma P-Tau181 levels in the cycling group only, confirming the effectiveness of moderate-intensity aerobic exercise in counteracting P-Tau181 accumulation in AD [116].

Noteworthily, Di Battista et al. subjected eleven healthy, active men to interval and high-intensity training on a cycle ergometer three times a week for 2 weeks, for a total of six training sessions [117]. Blood samples were collected before and after the intervention to assess the change in the concentration of plasma biomarkers, including Ng, NSE, T-Tau, VILIP-1, and brain-derived neurotrophic factor (BDNF). An increase in the plasma concentration of NSE, Ng, and BDNF was detected both after the first training session and after the last, highlighting the ability of exercise to influence their expression. Interestingly, T-Tau increased after the first training session, whereas no significant changes were found between the pre-and post-exercise phase of the last session [117].

Contrasting results were obtained by de Farias and colleagues by studying exercise-induced serum NSE changes in AD patients [118]. Specifically, 15 women diagnosed with AD underwent 22 physical/functional training sessions, including coordination, agility, balance, strength, and endurance activities, lasting 60 min per session, twice a week. In addition to improved judgment, problem-solving, and memory, the exercise programs significantly reduced serum NSE levels, demonstrating the efficacy of exercise in counteracting neurodegeneration and cognitive decline in AD [118]. Overall, although Olsson et al.’s meta-analysis found no significant plasma changes in NSE in AD patients, the studies by Di Battista and de Farias showed exercise-induced modulation, highlighting the need for further clarification.

The extraordinary power of exercise to regulate the expression of biomarkers of neuronal damage was confirmed by Desai et al. and Casaletto et al., who investigated the association between physical activity levels, Nfl concentrations, and cognitive decline in elderly subjects and subjects with frontotemporal lobar degeneration, respectively. Both studies reported that serum Nfl concentration was strongly influenced by physical activity, being lower in more active subjects. Furthermore, more active individuals were characterized by slower cognitive decline, demonstrating that greater physical activity leads to slower axonal degeneration [119,120]. However, these results are in contrast to more recent findings by Sewell et al., who found no exercise-induced modulation in plasma Nfl levels [114].

As suggested by Stojanovic and colleagues, a potential moderating factor of the effects of exercise in AD patients could be cardiovascular risk [121]. To test this hypothesis, the authors compared the levels of Ng, VILIP-1, SNAP-25, and Nfl in the CSF of clinically healthy subjects enrolled at the Knight Alzheimer Disease Research Center at Washington University with the aim of identifying and validating AD biomarkers. The study’s results claimed that neither Nfl nor SNAP-25 was modulated by exercise, while VILIP-1 and Ng were lower in subjects who engaged in exercise programs [121].

Finally, Yang et al. evaluated the effect of moderate-intensity aerobic exercise on elderly people with mild AD, dividing five volunteers with mild cognitive impairment into two groups: an aerobic exercise group subjected to cycling training at 70% of maximum intensity for 40 min a day, 3 days a week, for 3 months, and a control group. In addition to a marked cognitive improvement, a significant increase in Apo-A1 in the plasma of subjects in the aerobic exercise group after 3 months of intervention was detected, demonstrating the ability of this form of training to modulate the expression of this AD biomarker [122].

Unfortunately, evidence regarding the effects of exercise on the regulation of fluid biomarkers is still rather limited, suggesting the need for high-quality studies to confirm the efficacy of exercise in AD patients as well as the real diagnostic and/or prognostic power of fluid biomarkers.

Table 3 summarizes the main scientific evidence on the modulatory effects of exercise on biomarker levels in the CSF and plasma of patients with AD or other NDs.

## 5. Conclusions

Based on current knowledge, exercise emerges as the best non-pharmacological strategy to prevent and/or treat AD, counteracting neurodegeneration and cognitive decline [123,124,125]. Indeed, numerous RCTs have been conducted to determine the effects of exercise in AD patients, and although there is often considerable variability in the results obtained from the different studies, exercise overall seems to positively influence both physical and cognitive function [16]. Nevertheless, the full picture of the molecular mechanisms involved in brain adaptations to exercise in AD patients remains unclear and difficult to understand due to its complexity. Indeed, exercise is known to promote the expression of a wide variety of neurotrophic factors that regulate the function and vitality of neurons by promoting neurogenesis, but numerous other mechanisms are regulated by exercise [126,127]. Among these, autophagy and mitophagy may be partly responsible for the beneficial effects of exercise in AD patients, as they may promote Aβ turnover by limiting its accumulation in the brain [128,129]. Furthermore, the modulatory effects of exercise are known to include reducing oxidative stress and improving cerebral blood flow, two phenomena that play a crucial role in the development and progression of AD [130].

It is noteworthy thjat inflammation is also known to be associated with AD and undergoes exercise-induced regulation with beneficial effects in AD patients. Indeed, pro-inflammatory factors such as tumor necrosis factor α (TNF-α), caspase-1, and interleukin 1β (IL-β) have been linked to neurodegeneration in AD and their expression is increased in the brains of AD and MCI patients [131,132]. Interestingly, a crucial role of neuroinflammation in AD seems to be played by the conversion of microglia from the M1 phenotype, which is involved in pro-inflammatory processes and contributes to neurodegeneration, into the M2 phenotype, which has an anti-inflammatory function [133]. Not surprisingly, studies in different rodent models have shown that exercise can promote the polarization of microglia, in favor of the M2 phenotype, and ensure the development of an anti-inflammatory environment in the hippocampus by inducing the production of anti-inflammatory cytokines [15]. Taken together, all these exercise-regulated processes can establish a favorable environment for neuronal survival in AD patients and counteract neurodegeneration by modulating biomarker concentrations in body fluids accordingly. Indeed, the results of a systematic review with a meta-analysis conducted by Stigger and colleagues in 2019 showed how exercise can significantly reduce serum levels of interleukin-6 (IL-6) and TNF-α and positively modulate BDNF expression, overall suggesting a beneficial effect on neuronal viability [134].

Nevertheless, numerous unknowns still make it impossible to determine which form of exercise produces the best effects in AD patients. Indeed, the complexity of this pathology makes it necessary to consider numerous confounding variables when designing a study on the effects of exercise in AD patients. As suggested by Stojanovic and colleagues and described above, cardiovascular risk is a potential moderating factor in the effects of exercise in AD patients [121]. Furthermore, adequate social support from family members can significantly influence the patient’s psychological well-being and emotional state and provide the right individual motivation for rigorous exercise programs. Importantly, as suggested by Butt et al., *APOE* ε4 carriers, although cognitively intact, could be characterized by selective synaptic damage compared to non-carriers that could affect cognitive abilities and, consequently, the way such individuals cope with exercise programs [86]. Therefore, a successful strategy could be the design of individual exercise protocols, appropriately designed on the basis of the patients’ physical, psychic, emotional, and cognitive needs and characteristics, to identify the best forms and modes of exercise for a specific individual. However, the need for further investigation to define the modulatory function of exercise on biomarkers of neuronal damage is imperative, both to clarify its diagnostic and/or prognostic role and to determine its efficacy on neuronal and synaptic health.

One of the main limitations found in studies included in this critical review concerns the size of the study sample. Indeed, the difficulty of recruiting AD patients who meet the eligibility criteria of the study and who can exercise at a certain intensity and with regular frequency may result in the study population being narrowed down, with a consequent increase in effects due to variability. Another limitation found in some studies concerns the time of administration of the exercise program. Particularly, some studies evaluated the effects of a 6-month exercise program, which may not be sufficient to modulate the expression of the neuronal biomarkers addressed in this review. Finally, all the exercise protocols administered were significantly different from each other, not allowing for a comparison of the results obtained.

## Figures and Tables

**Table 1:** A schematic representation of the main evidence on AD biomarkers in the CSF.

Biomarker	Study Population	Biomarker Levels	Evidence	References
Aβ_42_ (pg/mL)	n = 24, age (years): 48–83 - 18 CN subjects; age (years): 48–83 - 4 patients with AD-type dementia; age (years): 73–81 - 2 patients with non-AD type dementia; age (years): 77	- CN subjects: 483–1071 in 15 subjects and 326–443 in 3 subjects - Patients with AD-type dementia: 230–426 - Patients with non-AD type dementia: 572–588	- Aβ_42_ reduction is associated with the presence of amyloid deposits in AD brains - Preclinical AD biomarker potential	[40]
P-Tau231 (pg/mL) P-Tau181 (pmol/L) P-Tau199 (fmol/mL)	n = 206 - 108 AD patients; age (years): 54–84; 65 females and 43 males - 22 DLB patients; age (years): 65–87; 7 females and 15 males - 24 FTD patients; age (years): 46–79; 15 females and 9 males - 7 VaD patients; age (years): 65–77; 4 females and 3 males - 22 OND patients; age (years): 49–81; 16 females and 6 males - 23 controls; age (years): 44–77; 14 females and 19 males	- AD patients: 667.5 for P-Tau231, 20.5 for P-Tau181, 1.7 for P-Tau199 - DLB patients: 213.5 for P-Tau231, 11.3 for P-Tau181, 1.1 for P-Tau199 - FTD patients: 86.5 for P-Tau231, 10.7 for P-Tau181, 1.1 for P-Tau199 - VaD patients: 201.0 for P-Tau231, 13.6 for P-Tau181, 1.3 for P-Tau199 - OND patients: 54.0 for P-Tau231, 11.3 for P-Tau181, 0.8 for P-Tau199 - Controls: 35.0 for P-Tau231, 11.0 for P-Tau181, 0.8 for P-Tau199	- Discriminates AD from other NDs - Predicts faster disease progression	[43]
P-Tau217 (pg/mL)	n = 753 - 290 CU Aβ+ patients; age (years): 63.8–77.9; 121 females and 169 males; 38.3% *APOE* ε4 carriers - 34 MCI Aβ− patients; age (years): 69.1–81.7; 12 females and 22 males; 20.6% *APOE* ε4 carriers - 47 MCI Aβ+ patients; age (years): 74.5–84.6; 24 females and 23 males; 46.8% *APOE* ε4 carriers - 6 patients with Aβ+ dementia; age (years): 80.4–85.7; 6 males; 33.3% *APOE* ε4 carriers - 376 CU Aβ− subjects; age (years): 62.9–78.7; 169 females and 207 males; 17.1% *APOE* ε4 carriers	- CU Aβ+ patients: 41.3–183.0 - MCI Aβ− patients: 52.8–114.6 - MCI Aβ+ patients: 98.3–484.8 - Patients with Aβ+ dementia: 201.7–558.4 - CU Aβ− subjects: 48.3–90.5	- Higher diagnostic sensitivity than P-Tau181 - Potential prognostic marker in MCI Aβ+ patients	[44]
Ng (pg/mL)	Clinical cohort: n = 116 - 30 AD patients; age (years): 78 ± 9; 15 females and 15 males - 32 AD patients with high T-Tau; age (years): 77 ± 9; 21 females and 11 males - 13 CJD patients; age (years): 68 ± 14; 8 females and 5 males - 11 non-AD individuals; age (years): 68 ± 6; 7 females and 4 males - 30 controls; age (years): 65 ± 12; 10 females and 20 males Cohort autopsy-confirmed: n = 147 - 50 AD patients; age (years): 76 ± 9; 22 females and 28 males; 34% *APOE* ε4 carriers - 47 non-AD individuals; age (years): 67 ± 11; 15 females and 32 males; 26% *APOE* ε4 carriers - 50 controls; age (years): 60 ± 6; 32 females and 18 males; 34% *APOE* ε4 carriers	Clinical cohort: - AD patients: 315–499 - AD patients with high T-Tau: 716–1148 - CJD patients: 703–1373 - non-AD patients: 319–699 - Controls: 193–306 Cohort confirmed by autopsy: - AD patients: 249–470 - non-AD patients: 137–416 - Controls: 193–370	- Increases in AD patients and with other NDs - Closely associated with T-Tau and P-Tau 181 in slowly progressive dementia, but not in CJD	[50]
NSE (ng/mL)	n = 63 - 32 AD patients; mean age (years): 74.37 ± 6.64; 21 females and 11 males - 32 controls; mean age (years): 50.75 ± 16.50; 14 females and 18 males	- AD patients: 18.12 - Controls: 8.46	- Indicates the presence of cognitive impairment - Direct correlation with T-Tau and P-Tau	[51]
Nfl (pg/mL)	Clinical cohort: n = 85 - 15 AD patients; age (years): 60–69; 7 females and 8 males - 11 bvFTD patients; age (years): 60–67; 2 females and 9 males - 4 CBS patients; age (years): 57–66; 1 female and 3 males - 19 DLB patients; age (years): 61–70; 5 females and 14 males - 10 HD patients; age (years): 44–60; 3 females and 7 males - 10 multiple sclerosis patients; age (years): 49–61; 6 females and 4 males - 6 semantic dementia patients; age (years): 56–68; 1 female and 5 males - 10 controls; age (years): 62–76; 3 females and 7 males	- AD patients: 700.0–1317.1 - bvFTD patients: 476.9–3714.2 - CBS patients: 1071.5–2698.5 - DLB patients: 703.3–1099.9 - HD patients: 2108.8–3218.4 - Multiple sclerosis patients: 523.3–932.3 - Semantic dementia patients: 1046.9–2039.4 - Controls: 417.9–735.6	Indicates non-AD-specific axonal damage	[57]
VILIP-1 (pg/mL)	n = 234 - 73 AD patients; mean age (years): 70 ± 8; 47 females and 26 males - 18 FTD patients; mean age (years): 68 ± 10; 9 females and 9 males - 26 PD patients; mean age (years): 70 ± 8; 11 females and 15 males - 20 ALS patients; mean age (years): 64 ± 13; 8 females and 12 males - 22 CJD patients; mean age (years): 65 ± 8; 14 females and 8 males - 75 controls; mean age (years): 69 ± 13; 45 females and 30 males	- AD patients: 119–220 - FTD patients: 81–134 - PD patients: 62–166 - ALS patients: 64–141 - CJD patients: 326–1173 - Controls: 72–119	- Positive correlation with T-Tau and negative correlation with Aβ_42_ - High in AD patients compared to controls - High in CJD patients	[59]
HFABP (ng/mL)	n = 295 - 66 AD patients; mean age (years): 75.4 ± 0.9; 41% females and 59% males; 71% *APOE* ε4 carriers - 139 MCI patients; mean age (years): 75.1 ± 0.7; 33% females and 67% males; 54% *APOE* ε4 carriers - 90 controls; mean age (years): 76.0 ± 0.6; 51% females and 49% males; 24% *APOE* ε4 carriers	- AD patients: 0.58 ± 0.03 - MCI patients: 0.54 ± 0.02 - Controls: 0.38 ± 0.03	- Reports brain atrophy in patients with low Aβ_42_ levels - Associated with neuronal lipid deregulation	[65]
ApoA-1 (mg/L)	n = 429 - 206 SCD patients; mean age (years): 61.0 ± 8.8; 42% females and 58% males; 42% *APOE* ε4 carriers - 223 MCI patients; mean age (years): 67.1 ± 8.2; 42% females and 58% males; 58% *APOE* ε4 carriers	- SCD patients: 3.4 ± 1.6 - MCI patients: 3.6 ± 1.9	High levels in patients with cognitive decline and *APOE* ε4 carriers	[68]
GAP-43 (pg/mL)	n = 93 - 39 CN Aβ- subjects; mean age (years): 72.8 ± 5.06; 29 females and 19 males - 33 CN Aβ+ subjects; mean age (years): 76.6 ± 6.36; 23 females and 10 males - 21 MCI patients or with Aβ+ dementia; mean age (years): 77.9 ± 7.06; 13 females and 8 males	- CN Aβ- subjects: 4780 ± 2220 - CN Aβ+ subjects: 5570 ± 3820 - MCI patients or with Aβ+ dementia: 5560 ± 3070	- Abundant in the CSF of AD patients - Predicts rapid accumulation of Aβ-related tau	[74]
YKL-40 (ng/mL)	n = 109 - 11 AD patients; mean age (years): 73.6 ± 5.6; 7 females and 4 males; 72.7% *APOE* ε4 carriers - 63 MCI patients; mean age (years): 73.8 ± 6.4; 18 females and 45 males; 54% *APOE* ε4 carriers - 35 controls; mean age (years): 75.9 ± 5.2; 20 females and 15 males; 22.9% *APOE* ε4 carriers	- AD: patients 467.1 - MCI patients: 374.2 - Controls: 335.0	Discriminates MCI patients from AD patients and predicts the conversion of MCI to AD	[78]
PSD-95 (pg/mL)	Initial cohort: n = 178 - 37 AD patients; 56–84; 16 females and 21 males - 62 patients with another neurodegeneration; age (years): 40–89; 34 females and 28 males - 59 neurocontrol patients; age (years): 20–85; 33 females and 26 males - 20 healthy controls; age (years): 23–77; 9 females and 11 males Validation cohort: n = 165 - 105 AD patients; age (years): 51–89; 45 females and 60 males - 6 patients with another neurodegeneration; age (years): 46–79; 2 females and 4 males - 33 neurocontrol patients; age (years): 25–84; 18 females and 15 males - 21 healthy controls; age (years): 21–85; 10 females and 11 males	Initial cohort: - AD patients: 452.6 ± 176.2 - Patients with another neurodegeneration: 316.1 ± 270.6 - Neurocontrol patients: 308.4 ± 267.9 - Healthy controls: 212.6 ± 70.1 Validation cohort: - AD patients: 279.1 ± 137.9 - Patients with another neurodegeneration: 129 ± 53.2 - Neurocontrol patients: 109.6 ± 37.3 - Healthy controls: 130.3 ± 67.7	Identifies the presence of synaptic neuronal damage	[81]
SNAP-25 (pg/mL)	Initial cohort: n = 178 - 37 AD patients; 56–84; 16 females and 21 males - 62 patients with another neurodegeneration; age (years): 40–89; 34 females and 28 males - 59 neurocontrol patients; age (years): 20–85; 33 females and 26 males - 20 healthy controls; age (years): 23–77; 9 females and 11 males Validation cohort: n = 165 - 105 AD patients; age (years): 51–89; 45 females and 60 males - 6 patients with another neurodegeneration; age (years): 46–79; 2 females and 4 males - 33 neurocontrol patients; age (years): 25–84; 18 females and 15 males - 21 healthy controls; age (years): 21–85; 10 females and 11 males	Initial cohort: - AD patients: 113.9 ± 30.3 - Patients with another neurodegeneration: 71.3 ± 24.1 - Neurocontrol patients: 91.0 ± 53.0 - Healthy controls: 83.2 ± 21.2 Validation cohort: - AD patients: 163.4 ± 61.6 - Patients with another neurodegeneration: 78.8 ± 29.5 - Neurocontrol patients: 88.3 ± 28.7 - Healthy controls: 96.9 ± 36.1	- Increases in AD patients compared to other neurodegenerations - AD-specific biomarker potential	[81]
NRG1 (pg/mL)	n = 162 - 54 AD patients; mean age (years): 69.4 ± 7.9; 33 females and 21 males; 64.7% *APOE* ε4 carriers - 20 MCI-AD patients; mean age (years): 70.2 ± 8.0; 12 females and 8 males; 38.9% *APOE* ε4 carriers - 31 patients with other MCI; mean age (years): 61.5 ± 9.6; 11 females and 20 males; 30.8% *APOE* ε4 carriers - 30 patients with other dementias; mean age (years): 68.7 ± 7.6; 11 females and 20 males; 46.4% *APOE* ε4 carriers - 27 controls; mean age (years): 62.0 ± 11.3; 23 females and 4 males; 16.0% *APOE* ε4 carriers	- AD patients: 364.7 ± 149.2 - MCI-AD patients: 342.6 ± 161.5 - Patients with other MCI: 304.9 ± 113.0 - Patients with other dementias: 287.5 ± 106.5 - Controls: 267.7 ± 104.2	Increases in AD patients and with other NDs	[88]

Aβ_42_: beta-amyloid 42; P-Tau: phosphorylated tau; Ng: neurogranin; NSE: neuron-specific enolase; Nfl: neurofilament light chain; VILIP-1: visinin-like protein 1; HFABP: heart fatty acid binding protein; ApoA-1: apolipoprotein A1; GAP-43: growth-associated protein 43; YKL-40: chitinase 3-like protein 1 or human cartilage glycoprotein 39; PSD-95: postsynaptic density protein 95; SNAP-25: synaptosomal-associated protein 25; NRG1: neuregulin 1; CN: cognitively normal; AD: Alzheimer’s disease; DLB: dementia with Lewy Bodies; FTD: frontotemporal dementia; VaD: vascular dementia; OND: other neurodegenerative disorders; CU Aβ+: cognitively normal beta-amyloid positive; MCI Aβ-: mild cognitive impairment beta-amyloid negative; MCI Aβ+: mild cognitive impairment beta-amyloid positive; CU Aβ-: cognitively normal beta-amyloid negative; CJD: Creutzfeldt–Jakob disease; bvFTD: behavioral variant of frontotemporal dementia; CBS: corticobasal syndrome; HD: Huntington’s disease; PD: Parkinson’s disease; ALS: amyotrophic lateral sclerosis; SCD: cognitive decline.

**Table 2:** A schematic representation of the main evidence on AD biomarkers in plasma.

Biomarker	Study Population	Biomarker Levels	Evidence	References
Aβ_42_ (pg/mL)	n = 249 Baseline: - 123 controls; mean age (years): 60.0 ± 7.1; 62 females and 61 males; 17.9% *APOE* ε4 carriers - 126 pre-AD patients; mean age (years): 59.0 ± 6.3; 63 females and 63 males; 41.3% *APOE* ε4 carriers Follow-up - 123 controls; mean age (years): 70.0 ± 7.1; 62 females and 61 males; 17.9% *APOE* ε4 carriers - 126 AD patients; mean age (years): 69.0 ± 6.3; 63 females and 63 males; 41.3% *APOE* ε4 carriers	Baseline: - Controls: 16.69 ± 3.84 - Pre-AD patients: 14.43 ± 3.96 Follow-up: - Controls: 15.33 ± 3.25 - AD patients: 9.59 ± 2.53	- Aβ_42_ reduction predicts the development of AD 8 to 10 years before disease onset	[91]
P-Tau231 (pg/mL) P-Tau181 (pg/mL) P-Tau217 (pg/mL)	n = 397 - 262 Aβ− subjects; mean age (years): 60.6 ± 4.45; 162 females and 100 males; 42.4% *APOE* ε4 carriers - 135 Aβ+ subjects; mean age (years): 62.2 ± 4.91; 81 females and 54 males; 76.3% *APOE* ε4 carriers	P-Tau231 - Aβ− subjects: 9.62 ± 4.33 - Aβ+ subjects: 15.0 ± 7.49 P-Tau181 - Aβ− subjects: 8.83 ± 3.21 - Aβ+ subjects: 11.0 ± 4.60 P-Tau217 - Aβ− subjects: 0.13 ± 0.055 - Aβ+ subjects: 0.18 ± 0.086	- Strong association with Aβ positivity detected by PET - P-Tau231 and P-Tau217 detect early brain changes associated with the presence of Aβ before the clinical manifestations of the disease	[97]
Ng (pg/mL)	n = 298 Discovery cohort: - 28 AD patients; mean age (years): 66 ± 6; 16 females and 12 males; 39.2% *APOE* ε4 carriers - 25 MCI patients; mean age (years): 65 ± 5; 13 females and 12 males; 28.0% *APOE* ε4 carriers - 29 controls; mean age (years): 63 ± 5; 15 females and 14 males; 17.2% *APOE* ε4 carriers Validation cohort: - 73 AD patients; mean age (years): 65 ± 6; 42 females and 31 males; 42.5% *APOE* ε4 carriers - 71 MCI patients; mean age (years): 66 ± 7; 39 females and 32 males; 31.0% *APOE* ε4 carriers - 72 controls; mean age (years): 64 ± 5; 37 females and 35 males; 19.4% *APOE* ε4 carriers	Discovery cohort: - AD patients: 250 ± 67 - MCI patients: 1567 ± 445 - Controls: 2010 ± 530 Validation cohort: - AD patients: 254 ± 69 - MCI patients: 1511 ± 390 - Controls: 2099 ± 540	Reduction in exosomes isolated from the blood of AD and MCI patients	[99]
Nfl (pg/mL)	n = 249 Baseline: - 123 controls; mean age (years): 60.0 ± 7.1; 62 females and 61 males; 17.9% *APOE* ε4 carriers - 126 pre-AD patients; mean age (years): 59.0 ± 6.3; 63 females and 63 males; 41.3% *APOE* ε4 carriers Follow-up - 123 controls; mean age (years): 70.0 ± 7.1; 62 females and 61 males; 17.9% *APOE* ε4 carriers - 126 AD patients; mean age (years): 69.0 ± 6.3; 63 females and 63 males; 41.3% *APOE* ε4 carriers	Baseline: - Controls: 10.71 ± 3.88 - Pre-AD patients: 13.24 ± 5.00 - Follow-up: - Controls: 11.90 ± 3.58 - AD patients: 16.17 ± 4.70	- Increased Nfl predicts the development of AD 8 to 10 years before disease onset	[91]
VILIP-1 (pg/mL)	n = 234 - 73 AD patients; mean age (years): 70 ± 8; 47 females and 26 males - 18 FTD patients; mean age (years): 68 ± 10; 9 females and 9 males - 26 PD patients; mean age (years): 70 ± 8; 11 females and 15 males - 20 ALS patients; mean age (years): 64 ± 13; 8 females and 12 males - 22 CJD patients; mean age (years): 65 ± 8; 14 females and 8 males - 75 controls; mean age (years): 69 ± 13; 45 females and 30 males	- AD patients: 24–36 - FTD patients: 21–42 - PD patients: 18–32 - ALS patients: 20–46 - CJD patients: 52–142 - Controls: 18–31	- High in AD patients compared to controls - High in CJD patients	[59]
HFABP (pg/mL)	n = 64 - 18 AD patients; age (years): 47–85; 13 females and 5 males - 14 CJD patients; age (years): 57–78; 8 females and 6 males - 16 DLB patients; age (years): 55–88; 12 females and 4 males - 16 controls; age (years): 32–76; 9 females and 7 males	- AD patients: 581–9029 - CJD patients: 1836–25,000 - DLB patients: 1292–25,000 - Controls: 445–3543	Increases in all NDs, especially in DLB patients	[102]
ApoA-1 (g/L)	n = 429 - 206 SCD patients; mean age (years): 61.0 ± 8.8; 42% females and 58% males; 42% *APOE* ε4 carriers - 223 MCI patients; mean age (years): 67.1 ± 8.2; 42% females and 58% males; 58% *APOE* ε4 carriers	- SCD patients: 1.4 ± 0.4 - MCI patients: 1.3 ± 0.3	- Low levels in patients with cognitive decline and *APOE* ε4 carriers	[68]
GAP-43 (pg/mL)	n = 298 Discovery cohort: - 28 AD patients; mean age (years): 66 ± 6; 16 females and 12 males; 39.2% *APOE* ε4 carriers - 25 MCI patients; mean age (years): 65 ± 5; 13 females and 12 males; 28.0% *APOE* ε4 carriers - 29 controls; mean age (years): 63 ± 5; 15 females and 14 males; 17.2% *APOE* ε4 carriers Validation cohort: - 73 AD patients; mean age (years): 65 ± 6; 42 females and 31 males; 42.5% *APOE* ε4 carriers - 71 MCI patients; mean age (years): 66 ± 7; 39 females and 32 males; 31.0% *APOE* ε4 carriers - 72 controls; mean age (years): 64 ± 5; 37 females and 35 males; 19.4% *APOE* ε4 carriers	Discovery cohort: - AD patients: 1996 ± 515 - MCI patients: 2372 ± 450 - Controls: 2738 ± 724 Validation cohort: - AD patients: 1926 ± 509 - MCI patients: 2325 ± 606 - Controls: 2722 ± 664	Reduction in exosomes isolated from the blood of AD and MCI patients	[99]
YKL-40 (ng/mL)	n = 145 - 41 mild AD patients; mean age (years): 75.04 ± 0.91; 33 females and 8 males - 20 moderate/severe AD patients; mean age (years): 74.55 ± 1.56; 16 females and 4 males - 49 MCI patients; mean age (years): 68 ± 1.00; 30 females and 19 males - 35 controls; mean age (years): 63.88 ± 0.96; 22 females and 13 males	- Mild AD patients: 407.81 ± 73.25 - Moderate/severe AD patients: 313.43 ± 68.72 - MCI patients: 176.49 ± 25.68 - Controls: 96.91 ± 11.02	- Increases in patients with early AD - Positive correlation with cognitive function in patients with mild AD	[105]
SNAP-25 (pg/mL)	n = 298 Discovery cohort: - 28 AD patients; mean age (years): 66 ± 6; 16 females and 12 males; 39.2% *APOE* ε4 carriers - 25 MCI patients mean age (years): 65 ± 5; 13 females and 12 males; 28.0% *APOE* ε4 carriers - 29 controls; mean age (years): 63 ± 5; 15 females and 14 males; 17.2% *APOE* ε4 carriers Validation cohort: - 73 AD patients; mean age (years): 65 ± 6; 42 females and 31 males; 42.5% *APOE* ε4 carriers - 71 MCI patients; mean age (years): 66 ± 7; 39 females and 32 males; 31.0% *APOE* ε4 carriers - 72 controls; mean age (years): 64 ± 5; 37 females and 35 males; 19.4% *APOE* ε4 carriers	Discovery cohort: - AD patients: 302 ± 80 - MCI patients: 575 ± 144 - Controls: 634 ± 166 Validation cohort: - AD patients: 489 ± 114 - MCI patients: 569 ± 152 - Controls: 628 ± 166	Reduction in exosomes isolated from the blood of AD and MCI patients	[99]
NRG1 (pg/mL)	n = 127 - 20 neurological controls; mean age (years): 60.6 ± 9.6; 14 females and 6 males - 19 non-AD MCI patients; 61.1 ± 8.4; 12 females and 7 males - 25 AD-MCI patients; 70.3 ± 5.8; 17 females and 8 males - 37 AD dementia patients; 67.7 ± 7.9; 23 females and 14 males - 26 non-AD dementia patients; 68.1 ± 7.0; 10 females and 16 males	- Neurological controls: 378.9 ± 400.7 - non-AD MCI patients: 488.4 ± 392.2 - AD-MCI patients: 707.6 ± 562.7 - AD dementia patients: 940.3 ± 737.5 - non-AD dementia patients: 615.5 ± 486.3	- High concentration in AD patients correlates with cognitive decline and synaptic damage	[107]

Aβ_42_: beta-amyloid 42; P-Tau: phosphorylated tau; Ng: neurogranin; Nfl: neurofilament light chain; VILIP-1: visinin-like protein 1; HFABP: heart fatty acid binding protein; ApoA-1: apolipoprotein A1; GAP-43: growth-associated protein 43; YKL-40: chitinase 3-like protein 1 or human cartilage glycoprotein 39; SNAP-25: synaptosomal-associated protein 25; NRG1: neuregulin 1; AD: Alzheimer’s disease; PET: positron emission tomography; MCI: mild cognitive impairment; FTD: frontotemporal dementia; PD: Parkinson’s disease; ALS: amyotrophic lateral sclerosis; CJD: Creutzfeldt–Jakob disease; DLB: dementia with Lewy Bodies; SCD: cognitive decline.

**Table 3:** A schematic representation of the main evidence for the influence of exercise on fluid biomarker levels.

Biomarker	Study Population	Exercise Protocol	CSF	Plasma	References
Aβ_42_	n = 85 cognitively normal adults; mean age (years): 64.31 ± 5.44; 52 females and 33 males; 42.4% *APOE* ε4 carriers	- Subjects wore an accelerometer for a week - The data collected were processed to calculate the time spent in light-, moderate-, or vigorous-intensity physical activity	High Aβ_42_ levels have been associated with moderate-intensity physical activity	/	[113]
	n = 99 cognitively unimpaired older adults - 32 control group: mean age (years): 68.7 ± 5.9; 19 females and 13 males; 28.1% *APOE* ε4 carriers - 34 moderate intensity group: mean age (years): 68.4 ± 4.2; 18 females and 16 males; 23.5% *APOE* ε4 carriers - 33 high intensity group: mean age (years): 70.2 ± 5.3; 17 females and 16 males; 27.3% *APOE* ε4 carriers	- Control group: 2-h information session on the benefits of exercise - Moderate-intensity group: cycling at constant intensity for 50 min (50–60% aerobic capacity; 13.0 Borg Scale) - High-intensity group: 10 min warm-up, 11 1 min intervals of intense exercise cycling at 18.0 Borg Scale, 80% aerobic capacity, interspersed with 2 min of active recovery, and a 9 min cool-down	/	No exercise-induced modulation	[114]
P-Tau181	n = 1108 cognitively normal adults; mean age (years): 61.1 ± 11.0; 461 females and 647 males; 161 *APOE* ε4 carriers	Of the total, 275 individuals self-reported via questionnaire that they practice regular physical activity at moderate intensity every day	Moderate daily physical activity is associated with a significant reduction in P-Tau181 levels	/	[115]
	n = 26 older adults with mild-to-moderate AD dementia - 18 cycling group; mean age (years): 76.8 ± 7.6; 7 females and 11 males; 100% *APOE* ε4 carriers - 8 stretching group; mean age (years): 79.3 ± 5.5; 2 females and 6 males; 100% *APOE* ε4 carriers	- Cycling group: cycling on recumbent stationary cycles for 20–50 min at moderate intensity (50–75% of HRR), 3 days/week for 6 months - Stretching group: seated movements and low-intensity static stretching (<20% of HRR), 3 days/week for 6 months	/	6 months of cycling slows down the plasma increase in P-Tau181 compared to the stretching group	[116]
Ng	n = 51 AD patients - 26 control group: mean age (years): 68.9 ± 8.05; 7 females and 19 males - 25 intervention group; mean age (years): 68.2 ± 6.94; 12 females and 13 males	Aerobic exercise on treadmill, stationary bike, and cross-trainer for 60 min/day, 3 days/week for 16 weeks, with moderate to high intensity	No modulation induced by moderate-to-high aerobic exercise	/	[112]
	n = 11 physically active adults; mean age (years): 28.8 ± 5.3; 11 males	HIIT on a bicycle ergometer (8–12 × 60 sec intervals at 100% of peak power output, interspersed by 75 sec recovery at 50 W) for 3 days/week for 2 weeks	/	Significant increase in plasma Ng levels after a single HIIT session	[117]
NSE	n = 11 physically active adults; mean age (years): 28.8 ± 5.3; 11 males	HIIT on a bicycle ergometer (8–12 × 60 sec intervals at 100% of peak power output, interspersed by 75 sec recovery at 50 W) for 3 days/week for 2 weeks	/	Significant increase in plasma NSE levels after a single HIIT session	[117]
	n = 15 AD patients, mean age (years): 68.3 ± 13.8; 15 females	22 training sessions (coordination, agility, balance, strength, and endurance activities), 60 min a day, 2 days a week, with a target effort intensity of 40–60% of the target heart rate	/	Exercise decreased plasma levels of NSE, reversing neuronal damage	[118]
Nfl	n = 51 AD patients - 26 control group: mean age (years): 68.9 ± 8.05; 7 females and 19 males - 25 intervention group; mean age (years): 68.2 ± 6.94; 12 females and 13 males	Aerobic exercise on treadmill, stationary bike, and cross-trainer for 60 min/day, 3 days/week for 16 weeks, with moderate-to-high intensity	No modulation induced by moderate-to-high aerobic exercise	/	[112]
	n = 160 individuals with autosomal dominant variants for FTLD; mean age (years): 50.7 ± 14.7; 84 females and 76 males	- Self-reported measure of physical activity by PASE over the last 7 days - Assessment of the weekly frequency and daily duration of the following recreational activities: walking; light, moderate, and strenuous sports; housework; gardening work; strength training	/	Strong association between higher reported physical activity and reduced plasma Nfl levels	[120]
	n = 99 cognitively unimpaired older adults - 32 control group: mean age (years): 68.7 ± 5.9; 19 females and 13 males; 28.1% *APOE* ε4 carriers - 34 moderate intensity group: mean age (years): 68.4 ± 4.2; 18 females and 16 males; 23.5% *APOE* ε4 carriers - 33 high intensity group: mean age (years): 70.2 ± 5.3; 17 females and 16 males; 27.3% *APOE* ε4 carriers	- Control group: 2-h information session on the benefits of exercise - Moderate-intensity group: cycling at constant intensity for 50 min (50–60% aerobic capacity; 13.0 Borg Scale) - High-intensity group: 10 min warm-up, 11 1 min intervals of intense exercise cycling at 18.0 Borg Scale, 80% aerobic capacity, interspersed with 2 min of active recovery, and a 9 min cool-down	/	No exercise-induced modulation	[114]
VILIP-1	n = 51 AD patients - 26 control group: mean age (years): 68.9 ± 8.05; 7 females and 19 males - 25 intervention group; mean age (years): 68.2 ± 6.94; 12 females and 13 males	Aerobic exercise on treadmill, stationary bike, and cross-trainer for 60 min/day, 3 days/week for 16 weeks, with moderate-to-high intensity	No modulation induced by moderate-to-high aerobic exercise	/	[112]
	n = 11 physically active adults; mean age (years): 28.8 ± 5.3; 11 males	HIIT on a bicycle ergometer (8–12 × 60 sec intervals at 100% of peak power output, interspersed by 75 sec recovery at 50 W) for 3 days/week for 2 weeks	/	No modulation in plasma VILIP-1 levels after a single HIIT session	[117]
ApoA-1	n = 50 mild AD patients: - 25 control group; mean age (years): 71.92 ± 7.28; 18 females and 7 males - 25 aerobic group; mean age (years): 72.00 ± 6.69; 15 females and 10 males	- Control group: no intervention, health education for 3 months - Aerobic group: cycling training at 70% of maximal intensity for 40 min/day, 3 day/week for 3 months	/	Aerobic exercise promoted a significant increase in plasma ApoA-1 levels in association with an improvement in cognitive function, mental state, and quality of life in mild AD patients	[122]
YKL-40	n = 51 AD patients - 26 control group: mean age (years): 68.9 ± 8.05; 7 females and 19 males - 25 intervention group; mean age (years): 68.2 ± 6.94; 12 females and 13 males	Aerobic exercise on treadmill, stationary bike, and cross-trainer for 60 min/day, 3 days/week for 16 weeks, with moderate-to-high intensity	No modulation induced by moderate-to-high aerobic exercise	/	[112]

Aβ_42_: beta-amyloid 42; P-Tau: phosphorylated tau; Ng: neurogranin; NSE: neuron-specific enolase; Nfl: neurofilament light chain; VILIP-1: visinin-like protein 1; ApoA-1: apolipoprotein A1; YKL-40: chitinase 3-like protein 1 or human cartilage glycoprotein 39; AD: Alzheimer’s disease; HRR: reserve heart rate; HIIT: high-intensity interval training; FTLD: frontotemporal lobar degeneration; PASE: physical activity scale for the elderly.

## Data Availability

No new data were created or analyzed in this study. Data sharing is not applicable to this article.
